# Δ9-Tetrahydrocannabinol Prevents Methamphetamine-Induced Neurotoxicity

**DOI:** 10.1371/journal.pone.0098079

**Published:** 2014-05-20

**Authors:** M. Paola Castelli, Camilla Madeddu, Alberto Casti, Angelo Casu, Paola Casti, Maria Scherma, Liana Fattore, Paola Fadda, M. Grazia Ennas

**Affiliations:** 1 Department of Biomedical Sciences, University of Cagliari, Cagliari, Italy; 2 Center of Excellence “Neurobiology of Addiction”, University of Cagliari, Cagliari, Italy; 3 National Institute of Neuroscience (INN), University of Cagliari, Cagliari, Italy; 4 CNR Institute of Neuroscience-Cagliari, National Research Council-Italy, Cittadella Universitaria di Monserrato, Cagliari, Italy; The Scripps Research Institute, United States of America

## Abstract

Methamphetamine (METH) is a potent psychostimulant with neurotoxic properties. Heavy use increases the activation of neuronal nitric oxide synthase (nNOS), production of peroxynitrites, microglia stimulation, and induces hyperthermia and anorectic effects. Most METH recreational users also consume cannabis. Preclinical studies have shown that natural (Δ9-tetrahydrocannabinol, Δ9-THC) and synthetic cannabinoid CB1 and CB2 receptor agonists exert neuroprotective effects on different models of cerebral damage. Here, we investigated the neuroprotective effect of Δ9-THC on METH-induced neurotoxicity by examining its ability to reduce astrocyte activation and nNOS overexpression in selected brain areas. Rats exposed to a METH neurotoxic regimen (4×10 mg/kg, 2 hours apart) were pre- or post-treated with Δ9-THC (1 or 3 mg/kg) and sacrificed 3 days after the last METH administration. Semi-quantitative immunohistochemistry was performed using antibodies against nNOS and Glial Fibrillary Acidic Protein (GFAP). Results showed that, as compared to corresponding controls (i) METH-induced nNOS overexpression in the caudate-putamen (CPu) was significantly attenuated by pre- and post-treatment with both doses of Δ9-THC (−19% and −28% for 1 mg/kg pre- and post-treated animals; −25% and −21% for 3 mg/kg pre- and post-treated animals); (ii) METH-induced GFAP-immunoreactivity (IR) was significantly reduced in the CPu by post-treatment with 1 mg/kg Δ9-THC1 (−50%) and by pre-treatment with 3 mg/kg Δ9-THC (−53%); (iii) METH-induced GFAP-IR was significantly decreased in the prefrontal cortex (PFC) by pre- and post-treatment with both doses of Δ9-THC (−34% and −47% for 1 mg/kg pre- and post-treated animals; −37% and −29% for 3 mg/kg pre- and post-treated animals). The cannabinoid CB1 receptor antagonist SR141716A attenuated METH-induced nNOS overexpression in the CPu, but failed to counteract the Δ9-THC-mediated reduction of METH-induced GFAP-IR both in the PFC and CPu. Our results indicate that Δ9-THC reduces METH-induced brain damage via inhibition of nNOS expression and astrocyte activation through CB1-dependent and independent mechanisms, respectively.

## Introduction

METH is an illicit, potent psychostimulant with neurotoxic properties [1]. The attention-enhancing properties of METH, its wide availability, its relative low cost, and its long-lasting psychoactive effects make it the most popular drug of the amphetamine-type stimulant (ATS) business, which accounted for 71% of global ATS seizures in 2011 [2]. METH abuse results in selective damage to both the dopaminergic (DAergic) and serotonergic (5-HTergic) terminals throughout the brain. Specifically, repeated administration of high doses of METH results in long-lasting alterations in markers of the DAergic and 5-HTergic systems, such as decreased levels of DA and 5-HT, tyrosine and tryptophan hydroxylase, DA, and 5-HT transporters [3], [4]. METH also increases the level of glial fibrillary acidic protein (GFAP), which is an index of gliosis and central nervous system injury and toxicity [5], [6]. Several cellular mechanisms underlying METH-induced neurotoxicity have been proposed, including blood-barrier breakdown, induction of inflammatory responses (microglial activation), apoptosis, DNA damage, and excitotoxic injury [7], [8].

Marijuana (*Cannabis sativa*) is frequently smoked by METH abusers [9], [10]; yet, whether it is smoked for the purpose of enhancing METH subjective' effects or attenuating its adverse effects (self-medication purposes) is unknown. While METH use has been linked to brain toxicity, marijuana smoking has been associated with both neurotoxic and neuroprotective effects [11], [12], [13]; whether the simultaneous use of METH and marijuana may accentuate or attenuate brain neurotoxicity has not yet been clearly defined. Regular cannabis abuse in METH-dependent adult subjects has been found to be associated with frontal, temporal, and striatal metabolic abnormalities compared to subjects solely using METH [14]; however, cannabis use was not found to exacerbate the neurotoxic effect of METH [10]. Decreased frontal N-acetylaspartate levels in the grey matter of adolescents co-abusing METH and marijuana has led to the hypothesis that concomitant heavy METH and marijuana use may induce neurotoxicity in the adolescent brain [15]. Adolescent METH and marijuana co-abusers also display increased regional striatal volume with respect to controls, with striatal volume positively correlated with the degree of METH exposure [16].

Although interactions between cannabinoid (CB1 and CB2) receptors and sensitivity to METH have been reported [17], their interaction in neurotoxicity has been scarcely investigated. We have recently showed that exposure to a neurotoxic METH treatment results in the sustained up-regulation of CB1 receptor expression across certain key brain regions implicated in the regulation of emotional and cognitive responses, including the medial prefrontal cortex, striatum, basolateral amygdala, and hippocampal formation [18]. Several studies have provided compelling evidence for the neuroprotective effects of cannabinoid CB1 receptor agonists in several models of neuronal injury [19]. Extensive *in vitro* and *in vivo* studies have shown that natural cannabinoids, e.g. Δ9-tetrahydrocannabinol (Δ9-THC) and cannabinol, and synthetic CB1 receptor agonists, can attenuate experimentally-induced neurotoxicity in multiple pathological conditions, such as glutamate excitoxicity, hypoxia, ischemic stroke, brain trauma, and oxidative stress [20]–[23]. The neuroprotective effects of natural and synthetic cannabinoids have been also shown animal models of Alzheimer's disease [24], Parkinson's disease [25], Huntington's disease [26], and multiple sclerosis [27]. Indeed, a local and temporary increase of 2-arachidonoylglycerol (2-AG) level in response to traumatic brain injury has also been established [28].

Converging evidence suggests that increased production of NO plays a role in METH-induced neurotoxicity, as METH-administration increases neuronal nitric oxide synthase (nNOS) activity and increases levels of nitrates and peroxynitrites [29], [30]. Mice lacking the nNOS gene are significantly more resistant to N-Methyl-D-aspartate- (NMDA) or METH-induced neurotoxicity and 3-nitrotyrosine production than wild-type mice [31], [32]. Notably, nNOS plays also an important role in cannabinoid-induced neurogenesis and neuroprotection through both the CB1 and CB2 receptors [33], [34]. Few studies have reported that Δ9-THC prevents 3,4-methylenedioxymethamphetamine(MDMA) neurotoxicity [35], [36]; yet, no study has thus far investigated the effects of cannabinoids in METH-induced neurotoxicity.

In the present study, we evaluated the neuroprotective effects of Δ9-THC in an animal model of METH neurotoxicity. We used a METH treatment protocol previously shown to readily induce neurotoxicity [3], [8], which includes using a binge administration of high dose of METH. Specifically, rats receiving a neurotoxic regimen of subcutaneous (s.c.) METH administrations (4×10 mg/kg, 2 hours apart) were pre-treated (PRE) or post-treated (POST) with Δ9-THC administered intraperitoneally (i.p.) at the dose of 1 or 3 mg/kg at room temperature, and were then sacrificed 3 days after the last METH administration. To determine the role of the CB1 receptor on Δ9-THC treatment, rats were pretreated (i.p.) with the CB1 receptor antagonist/inverse agonist SR141716A (SR 1 mg/kg) 15 min before each injection of Δ9-THC (1 mg POST). The neuroprotective effects of Δ9-THC were determined by examining the reduction of reactive astrogliosis (through GFAP-immunostaining) and the expression of nNOS, which are both altered in METH-induced neurotoxicity. To our knowledge, this is the first study specifically aimed at elucidating whether either a previous (PRE) or a later (POST) Δ9-THC exposure may affect METH-induced neurotoxicity.

## Materials and Methods

### Ethical statement

All procedures involving animals and their care were carried out in an animal facility according to Italian (D.L. 116/92 and 152/06) and European Council directives (609/86 and 63/2010) and in compliance with the approved animal policies by the Ethical Committee for Animal Experiments (CESA, University of Cagliari) and the Italian Department of Health. Specifically, all protocols used in the present study have been approved by the CESA, University of Cagliari (permit number 5/2011). Animals were monitored continuously during the drug treatment, i.e. every 30 min from 7.00 am to 9 pm. All rats were perfused under deep anesthesia with chloral hydrate and all efforts were made to minimize suffering.

### Animals

A total of 109 adult male Sprague-Dawley rats (300–350 g; Charles River, Como, Italy) were used in this study. Animals were individually housed at a temperature of 22°C with 60% humidity under a 12 h light/dark cycle (lights on from 7:00 a.m.). The compliance to aspects of animal welfare law was regularly monitored by the veterinary staff.

### Drugs and experimental procedures

(+)Methamphetamine hydrochloride (METH, Sigma-Aldrich, MO, USA), was diluted in sterile saline and administered subcutaneously (s.c.) at a volume of 1 mL/kg. Δ9-THC (RTI International, Research Triangle Park, NC, USA), 50 mg/mL in ethanol, and SR141716A (SR, kindly provided by Sanofy-Synthelabo, Montpellier, France) were dissolved in Tween 80 (2%), ethanol (2%), and saline (96%), and administered intraperitoneally (i.p.) at a volume of 1 mL/kg.

Rats were randomly distributed into 2 groups receiving four s.c. administrations of either 10.0 mg/kg METH (calculated as free base, n = 81) or saline (SAL, n = 28) at 2 h intervals. METH doses and treatment were selected on the basis of their ability to induce neurotoxic effects on both serotoninergic and dopaminergic systems, and to induce lasting neuronal damage comparable to that detected in METH users [3], [8].

**Figure 1:**
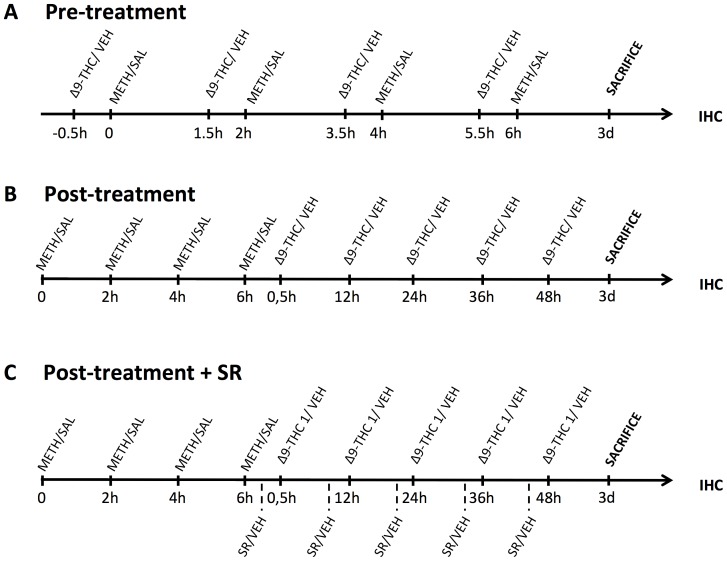
Synopsis of the experimental design, including treatment schedule and IHC assays. A. Pre-treatment: rats received injections of Δ9-THC (1 or 3 mg/kg) or vehicle (VEH) 30 min before each METH or SAL injection, and 3 days (3d) after the last METH or SAL injection were perfused and used for IHC analysis. B. Post-treatment: rats received injections of Δ9-THC (1 or 3 mg/kg) or vehicle (VEH) 0.5, 12, 24, 36 and 48 h after the last METH or SAL administration, and 3 days (3d) after the last METH injection were perfused and used for IHC analysis. C. Post-treatment + SR treatment: rats received injection of SR (1 mg/kg, i.p.) or VEH 15 min prior each Δ9-THC (1 mg/kg) or VEH post-treatment injection, and 3 days (3d) after the last METH or SAL injection were perfused and used for IHC analysis. 0, 2 h, 4 h, 6 h: 1st, 2nd, 3rd and 4th injection of METH (10 mg/kg, s.c.) or SAL; IHC: immunohistochemistry; SR: SR141716A; VEH: vehicle.

As illustrated in Figure 1, METH- and SAL-treated rats received injections of Δ9-THC (1 or 3 mg/kg) or vehicle (VEH, 1 mL/kg) 30 min before (pre-treatment group, PRE, Fig. 1A) or 0.5, 12, 24, 36 and 48 h after the last METH or SAL administration (post-treatment group, POST, Figure 1B). Different groups of METH- and SAL-treated animals were pretreated (i.p.) with SR (1 mg/kg) or VEH administered 15 min before each injection of 1 mg/kg Δ9-THC or VEH (post-treatment + SR group, Figure 1C).

### Temperature and body weight

A digital thermometer was used to measure rectal temperature before the first injection of METH and 1 h after each successive drug injection. The body weight of animals was measured immediately before the first injection of METH and 24 h after.

During the treatment with METH, when the body temperature of the rats reached 40°C, they were cooled by moving them in a cage with ice.

### Brain tissue preparation and nNOS and GFAP immunofluorescence staining

Three days after the last METH injection, rats were deeply anaesthetized with chloral hydrate (400 mg/kg, i.p.), and transcardially perfused with 4% paraformaldehyde and 0.1% glutaraldehyde in 0.1 M phosphate-buffered saline (PBS, pH 7.4). Brains were rapidly removed and post-fixed in the same fixative for 6 h. After repeated washing in 0.1 M PBS, brains were cryoprotected in 30% sucrose in PBS for 48 h. Immunostaining was performed on free-floating coronal sections (thickness: 40 µm) which were obtained using a cryostat at levels comprising the brain areas selected for this study. To facilitate the identification of the selected brain areas, adjacent sections were also collected and stained with Neutral Red. We performed pre-blocking of tissue sections using normal goat serum (NGS, 10%), bovine serum albumin (BSA, 1%) and Triton X-100 (0.2%) in PBS for 1 h at room temperature. As concerns GFAP-immunofluorescence single-labeling, we used a mouse monoclonal anti-GFAP antibody (1:5000; Millipore Temecula, CA, USA) in PBS containing 0.2% Triton X-100, 0.1% BSA, and 1% NGS to incubate sections for 48 h at 4°C. Then, we washed sections in PBS containing 0.2% Triton X-100 and incubated them with Alexa Fluor 594-labeled goat anti-mouse IgG (1:400; Molecular Probes, Eugene, OR, USA) for 1 h in the dark at room temperature.

For nNOS-immunofluorescence single-labeling, sections were incubated for 48 h at 4°C with a rabbit polyclonal anti-neuronal nitric oxide synthase antibody (1∶3000; Millipore, Temecula, CA, USA) in PBS containing 0.2% Triton X-100, 0.1% BSA, and 1% NGS. After washing sections in PBS containing 0.2% Triton X-100, sections were incubated with Alexa Fluor 488-labelled goat anti-rabbit IgG (1:400; Molecular Probes, Eugene, OR, USA) for 1 h in the dark at room temperature.

Finally, all sections were rinsed and mounted on slides using VectaShield anti-fade mounting media (Vector Inc.). We performed standard control experiments by omitting either the primary or secondary antibody; no cellular labeling was yielded.

### GFAP and nNOS immunofluorescence staining: imaging and quantitative analysis

An Olympus IX 61 microscope, furnished with 2.5, 4, 10, 20 and 60× planapochromatic oil immersion objectives, was used for observations. An Olympus 12-bit cooled F View II camera (Hamburg, Germany) was used for capturing the images Excitation light was attenuated with a 6% transmittance neutral density filter.

For each animal, analysis of nNOS-immunoreactivity (IR) neurons and GFAP-IR was performed on one tissue section out of every 3 successive sections, for a total of 8 and 12 sections containing the cingulate cortex areas 3 and 1 (Cg3 and Cg1) and the caudate-putamen (CPu), respectively. The total size of the examined area in which nNOS-IR neurons and GFAP-IR were counted was chosen according to the extension of the region under analysis, in order to include almost the whole area (either Cg3 and Cg1 or CPu). According to the atlas of Paxinos and Watson [37], the selected coronal levels of these sections corresponded to the levels of plates 6–8 for the Cg3 and Cg1 (AP: +4.20 to +3.2) and 11–29 for the CPu (AP: +1.70 to −0.30).

We carried out semi-quantitative analysis of GFAP using the 20× objective on 3 non overlapping regions of interest (ROIs, roughly 140000 µm^2^) from one out of every 3 slices of the targeted brain region (Cg3, Cg1 or CPu). The focus depth was extended by summing the maximum intensity of several images taken at focus steps of 0.25 µm depth intervals to a total of 2 µm thickness using the Z-stack module (Olympus Soft Imaging Solution, GNHB, Munster, Germany). After capture, images were analyzed using the Cell P AnalySIS software module. Density thresholding to the single channel grey scale images was applied to detect positively stained fibers. Subsequently, for each image we estimated the proportion (%) of area occupied by fibers, and for each animal we calculated average values from images of all tissue sections. The number of nNOS positive cell bodies was counted bilaterally in 8 (Cg3, Cg1) and 12 (CPu) sections per animal. In these sections, 6 non-overlapping randomly selected ROIs of 0.15 mm^2^ were examined with a 20× objective by two trained observers blind to drug treatment. Limits of the ROI were defined based on structural details within the tissue sections to ensure the ROIs did not overlap. The distance among the 6 ROIs was superior to 40 µm to avoid overlapping; 20 µm was the averaged diameter of neurons on the ROI. nNOS positive cells touching the inferior or the right sides of the ROI were excluded from counting. The number of nNOS-IR neurons was expressed as mean/mm^2^ ± SEM.

### Statistical analysis

Densitometric data were calculated as means ± SEM. Data from METH- and SAL-treated rats were compared using two-tailed unpaired Student's t-tests. Data from METH-Δ9-THC-treated and METH-VEH-treated rats were analyzed by two-way analysis of variance (ANOVA) with treatment (Δ9-THC doses vs VEH) and time of treatment (Pre- vs Post-METH administration) as factors, followed by lower-order ANOVAs where appropriate. Repeated measures ANOVA was used for body weight and temperature measurements. *Post-hoc* comparisons were performed with the Bonferroni test. Alpha was set at *p* = 0.05. METH-induced mortality was analyzed using Fisher's test.

## Results

### Effects of METH on body temperature and weight

**Figure 2:**
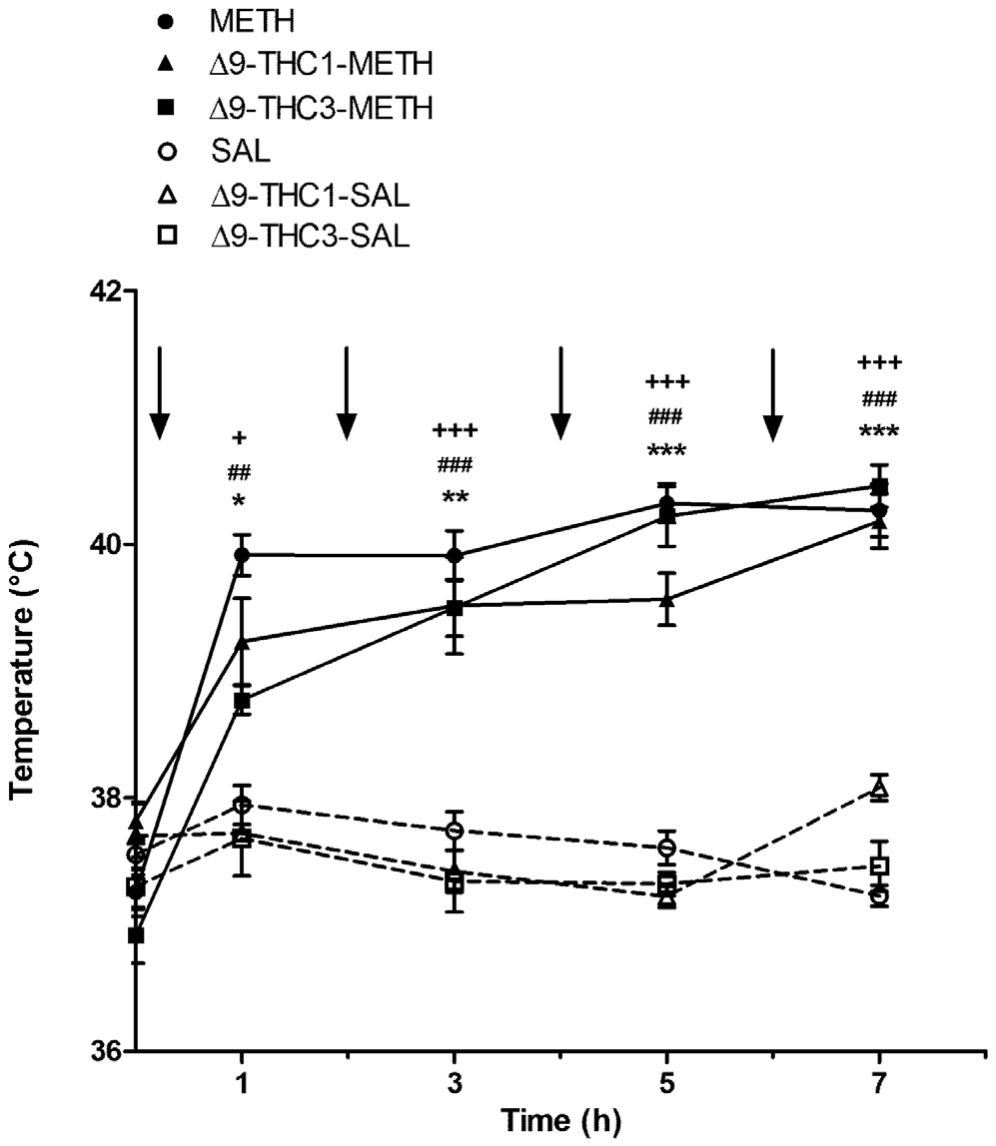
Core body temperature: effect of methamphetamine (METH) in the presence and absence of Δ9-THC (1 and 3 mg/kg). Rats were given SAL (1 mL/kg) or METH (4×10 mg/kg s.c., every 2 h) with and without Δ9-THC (1 and 3 mg/kg) pre-treatment. Body temperature was measured prior to and 1 h after each METH injection. Values are expressed as means ± SEM. Arrows indicate each injection of METH or SAL. No difference in baseline temperature was detected among groups. METH administration resulted in a significant increase in rectal temperature over time in comparison with SAL-treated rats. Both doses of Δ9-THC did not significantly change rectal temperature in METH-administered rats at any time point. METH: *p<0.05, **p<0.01 and ***p<0.001 vs corresponding SAL group at each time point. Δ9-THC1-METH: **^##^**p<0.01 and **^###^**p<0.001 vs corresponding Δ9-THC1-SAL group at each time point; Δ9-THC3-METH: **^+^**p<0.05 and **^+++^**p<0.001 vs corresponding Δ9-THC 3-SAL group at each time point.

Drug-induced alterations in body temperature were analyzed by two-way repeated-measures ANOVA with treatment and time as factors (Figure 2). Repeated METH injections induced rapid and significant hyperthermia in a time-dependent fashion [treatment: F_(1,35)_ = 82.3, *p*<0.0001; time: F_(4,140)_ = 43.8, *p*<0.0001; treatment x time interaction: F_(4,140)_ = 32.6, *p*<0.0001]. No significant difference was observed between METH and SAL-treated rats at baseline (SAL: 37.57±0.10; METH: 37.26±0.13). Rectal temperature was increased immediately after the first injection with the maximal hyperthermic effect observed after the third and fourth METH administration. Bonferroni *post-hoc* test showed that rats receiving METH had significantly higher temperatures compared to SAL-treated rats at all time points (1 h: *p*<0.05; 3 h: *p*<0.01; 5 h and 7 h: *p*<0.0001).

Moreover, the effect of Δ9-THC pre-treatment on body temperature in METH- and SAL-treated rats was analyzed separately for Δ9-THC 1 and Δ9-THC 3 mg/kg by two-way repeated-measures ANOVA with treatment and time as factors. There was no difference between groups in the basal temperature (Δ9-THC1-SAL =  37.70±0.27; Δ9-THC1-METH =  37.82±0.13; Δ9-THC3-SAL =  37.30±0.23; Δ9-THC3-METH =  36.92±0.22). METH administration induced a significant hyperthermia in a time-dependent fashion in both Δ9-THC1- and VEH-pretreated rats [treatment: F_(1,36)_ = 60.7, *p*<0.0001; time: F_(4,36)_ = 11.7, *p*<0.0001; treatment x time interaction: F_(4,36)_ = 10.1, *p*<0.0001] and Δ9-THC3- and VEH-pretreated rats [treatment: F_(1,36)_ = 81.7, *p*<0.0001; time: F_(4,36)_ = 33.2, *p*<0.0001; treatment x time interaction: F_(4,36)_ = 31.8, *p*<0.0001]. As shown in Figure 2, rats receiving Δ9-THC1-METH and Δ9-THC3-METH had significantly higher temperatures compared to Δ9-THC1-SAL and Δ9-THC3-SAL at all time points. Pre-treatment with both doses (1 and 3 mg/kg) of Δ9-THC did not significantly reduced body temperature in METH-administered rats.

In agreement with our previous findings [18], METH-treated rats showed a significant (*p*<0.0001) decrease in body weight (−10%) 24 h after the first administration, whereas no change in body weight was observed in SAL-treated rats. As previously described [3], METH-induced mortality rate was approximately 27%.

### Effects of METH on nNOS and GFAP immunoreactivity (IR)

**Figure 3:**
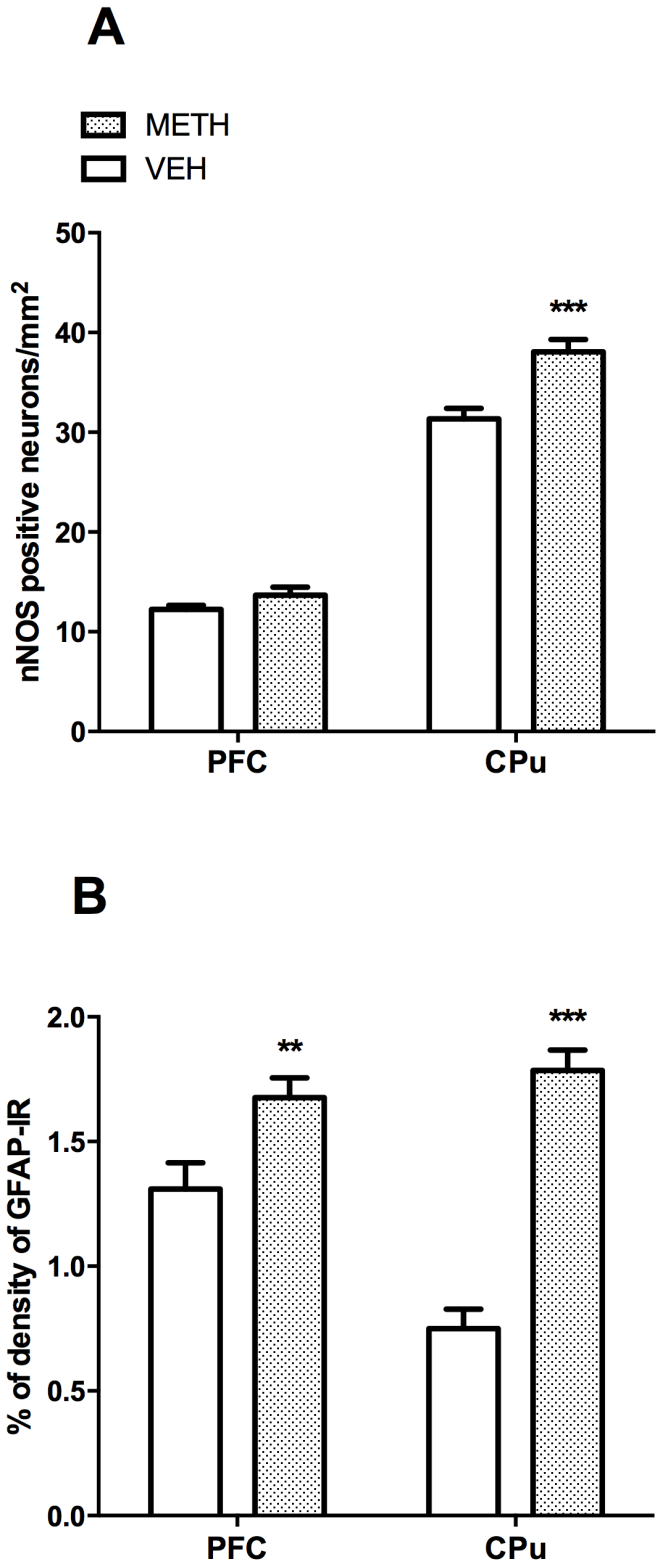
METH increases the number of neuronal nitric oxide synthase (nNOS) neurons and GFAP-immunoreactivity (IR). Values represent means ± SEM of either number of nNOS positive neurons, expressed per mm^2^ (A) or as percentage of GFAP-IR density (B). **p<0.01 and ***p<0.001 compared to SAL.

Consistent with prior studies implicating nNOS over-expression and astroglial reaction in METH-induced neurotoxicity [6], [29], in the present study, rats treated with a neurotoxic regimen of METH showed increased expression of GFAP in the CPu and PFC and of nNOS in the CPu (Figure 3). Specifically, METH administration significantly increased the number of striatal nNOS-positive cells (t_(29)_ = 4.02, *p*<0.001; +21%, Figure 3A) and GFAP-IR levels in the CPu and PFC (t_(20)_  = 9.06, *p*<0.0001, t_(24)_ = 2.83, *p*<0.01; +137% and +27%, respectively) as compared to saline administration (Figure 3B). No difference was observed in the number of nNOS positive cells in the PFC (Figure 3A).

### Δ9-THC attenuates the METH-induced increase of nNOS expression and GFAP-IR

We then evaluated the effect of Δ9-THC on METH-induced neurotoxicity. When administered alone to SAL-treated rats, Δ9-THC did not alter nNOS expression in the CPu (SAL-VEH = 31.36±1.04; SAL-Δ9-THC 1 = 31.60±0.98; SAL-Δ9-THC 3 = 30.40±1.25), nor GFAP-IR in the CPu (SAL-VEH = 0.75±0.08; SAL-Δ9-THC 1 = 0.80±0.08; SAL-Δ9-THC 3 = 0.87±0.07), nor GFAP-IR in the PFC (SAL-VEH = 1.34±0.11; SAL-Δ9-THC 1 = 1.26±0.09; SAL-Δ9-THC 3 = 0.96±0.07).

**Figure 4:**
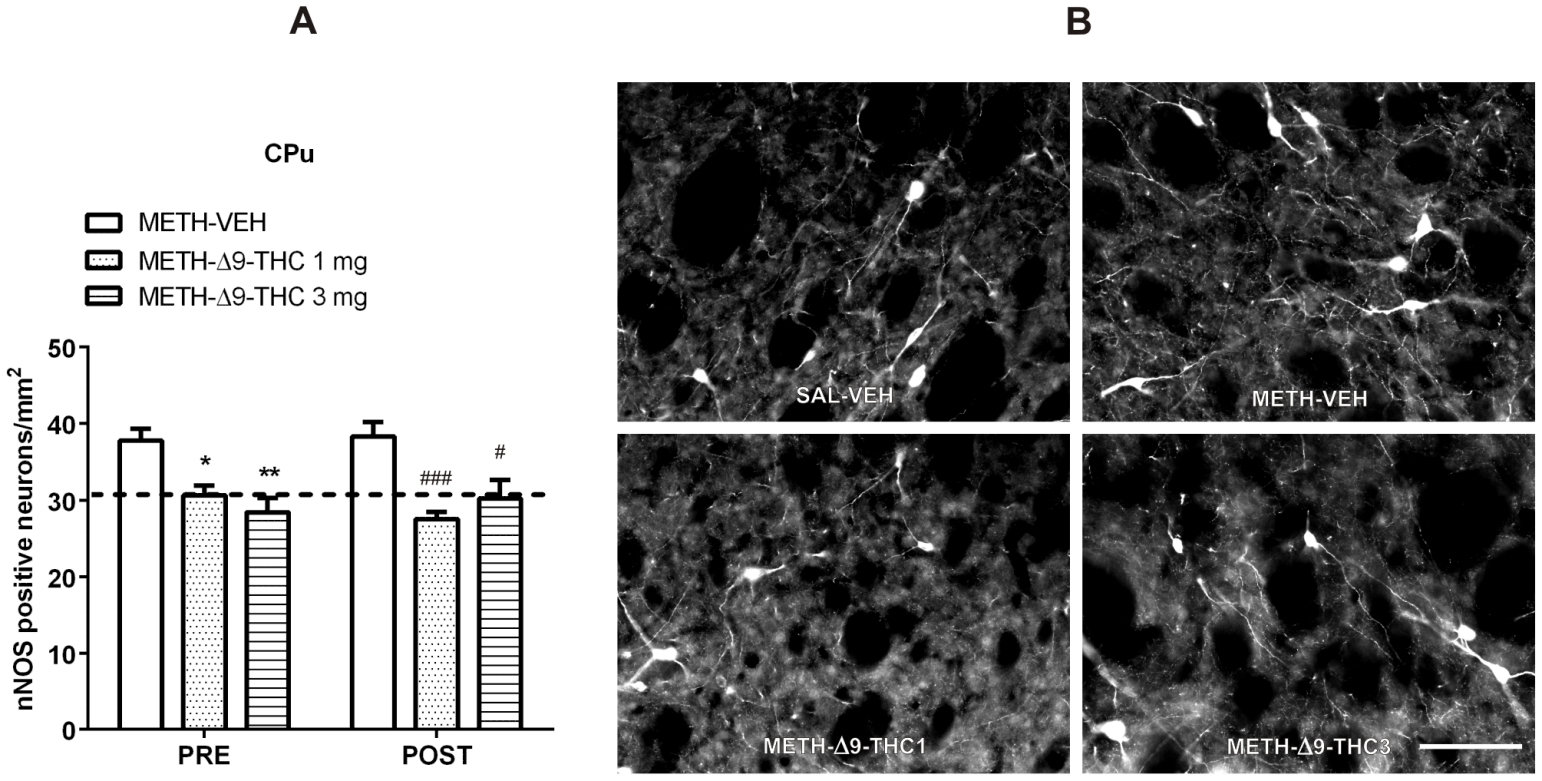
Δ9-THC reduces METH-induced increase of nNOS neurons in the CPu. A. Rats received injections of 1 or 3/kg of Δ9-THC either 0.5 h before each METH injection (PRE) or 0.5, 12, 24, 36, and 48 h after the last METH administration (POST), and were sacrificed 3 days after the last METH injection. Pre- and Post-treatment with both doses of Δ9-THC significantly decreased the number of nNOS positive neurons in the CPu. *p<0.05 and **p<0.01 vs PRE METH-VEH; **^#^** *p*<0.05 and **^###^** *p*<0.001 vs POST METH-VEH (Bonferroni's *post-hoc* test). Horizontal dot lines represent the values of nNOS positive neurons (31±1.03) in SAL-VEH group. B. Representative images of nNOS immunohistochemical staining 72 h after the last METH or SAL administration in SAL-VEH, METH-VEH, METH-Δ9-THC 1 and 3 mg. Scale bar  =  100 µm.

Regarding nNOS expression in METH-treated rats (Figure 4), two-way ANOVA [factors: time of treatment (pre- and post-METH administration) and treatment (VEH, Δ9-THC 1 mg/kg, Δ9-THC 3 mg/kg) showed a main effect of treatment in the CPu [F_(2,37)_  = 20.53, *p*<0.0001], resulting in a lower expression of nNOS in Δ9-THC (1 and 3 mg/kg)-treated than in VEH-treated rats (*p*<0.001, Bonferroni test). No effect of time [F_(1,37)_ = 0.031, *p* = 0.86] or treatment x time interaction [F_(2,37)_  = 1.032, *p* = 0.366] were observed.

To better evaluate the effect of Δ9-THC treatment, data were analyzed separately for time of treatment (pre- and post-METH administration) by one-way ANOVA, followed by the *post-hoc* Bonferroni test. As shown in Figure 4, pre- and post-treatment of both doses of Δ9-THC significantly decreased the number of nNOS positive neurons in the CPu. In particular, compared with VEH-treated groups, pre-treatment with Δ9-THC (PRE, 1 and 3 mg/kg) displayed a significant decrease of nNOS positive neurons by −19% and −25%, respectively, while post-treatment with Δ9-THC (POST, 1 and 3 mg/kg) decreased nNOS labelled neurons by −28% and −21%, respectively. No evidence for a dose-response effect of Δ9-THC treatment was observed. Taken together, these data indicate that Δ9-THC attenuated the neurotoxic effect of METH (Figure 4).

**Figure 5:**
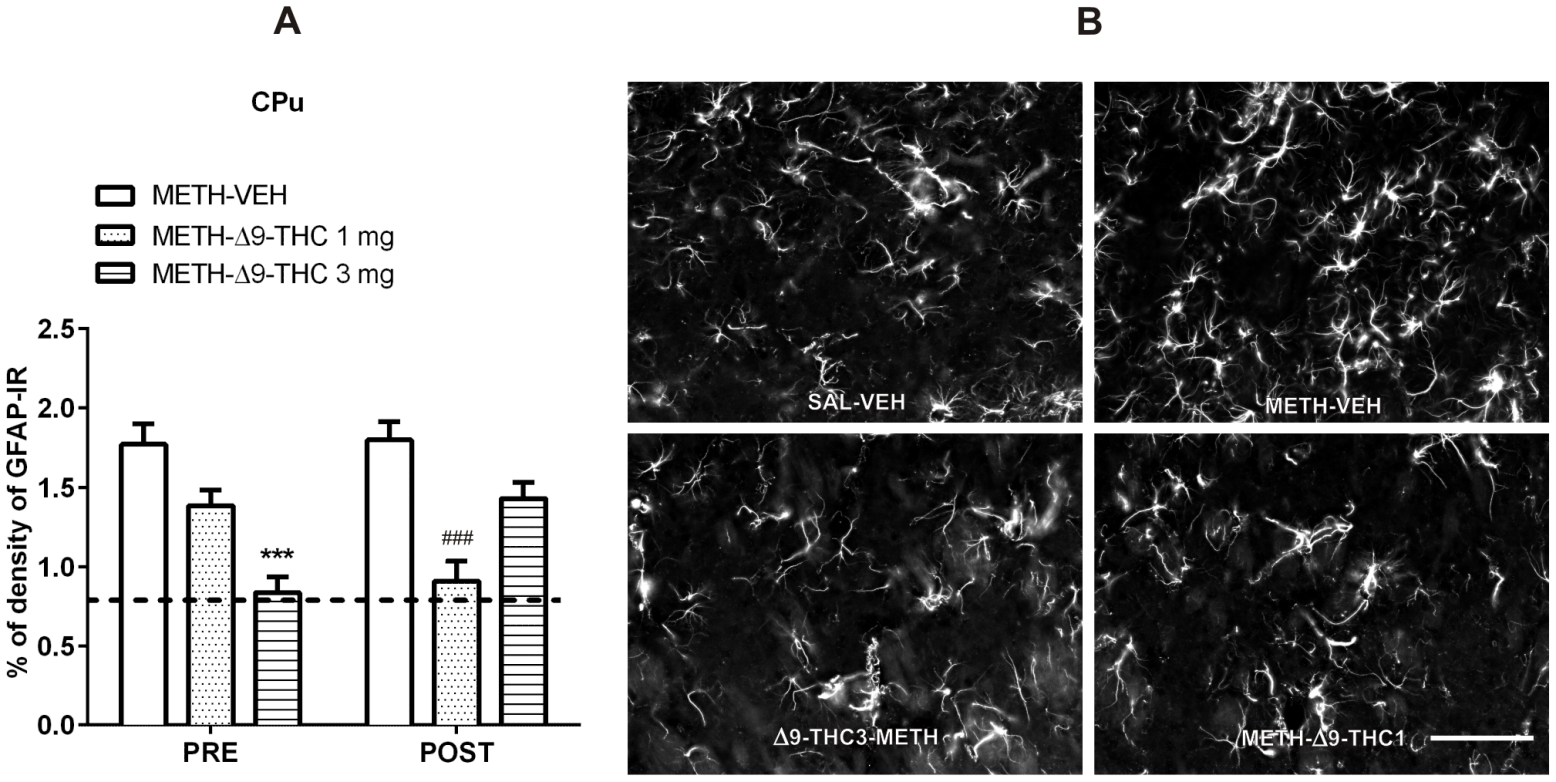
Δ9-THC reduces METH-induced astrogliosis in the CPu. A. Rats were treated as described in the legend of Figure 4. Two-way ANOVA revealed a significant effect of treatment (F_(2,32)_ = 16.28, *p*<0.0001) as well as a significant interaction between time of treatment and treatment (F_(2,32)_ = 8.12, *p* = 0.0014). *Post-hoc* comparisons showed that GFAP-IR was lower in the CPu of Post Δ9-THC (1 mg/kg) and Pre Δ9-THC (3 mg/kg) treated rats than in controls (METH-VEH). ***p<0.001 vs PRE METH-VEH and **^###^** *p*<0.001 vs POST METH-VEH (Bonferroni's *post-hoc* test). Horizontal dot lines represent the values of percentage of GFAP-IR density (0.75±0.07) in SAL-VEH group. B. Representative images of GFAP immunostaining in the CPu 72 h after the last METH or SAL administration in SAL-VEH, METH-VEH, METH-Δ9-THC 1 and 3 mg. Scale bar  =  100 µm.

As concerns METH-induced activation of astrocytes, in the CPu (Figure 5), a two-way ANOVA [factors: time of treatment (Pre- and Post-METH administration) and treatment (VEH, Δ9-THC 1 mg/kg, Δ9-THC 3 mg/kg)] detected a significant effect of treatment [F_(2,32)_ = 16.28, *p*<0.0001] and a treatment x time interaction [F_(2,32)_ = 8.12, *p*<0.01]. Bonferroni *post-hoc* comparisons showed that GFAP-IR was significantly lower in the CPu of Δ9-THC3 pre-treated (−53%, *p*<0.001) and Δ9-THC1 post-treated rats (−50%, *p*<0.001) than in corresponding control groups (PRE and POST METH-VEH rats; Figure 5).

**Figure 6:**
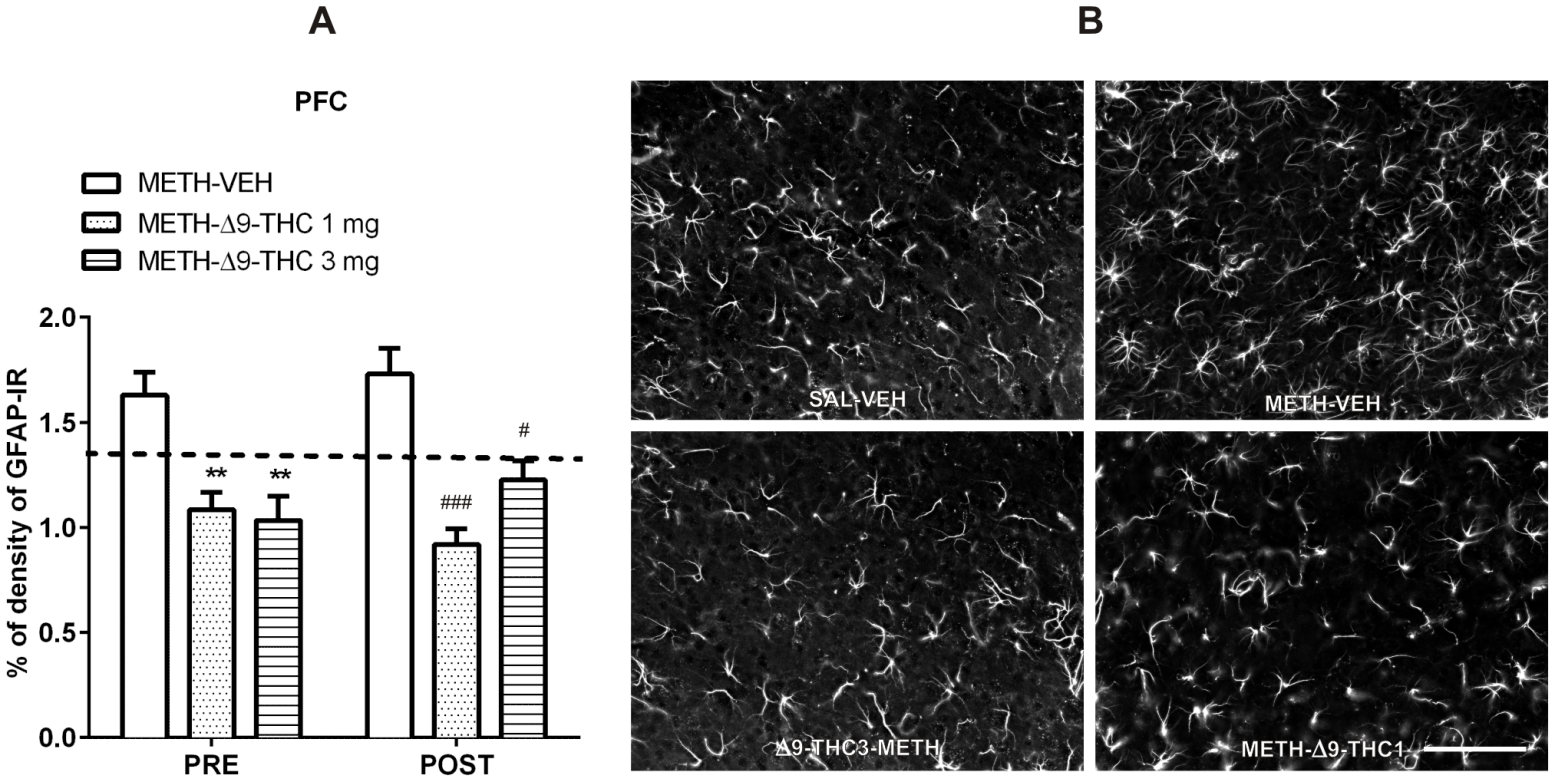
Δ9-THC reduces METH-induced astrogliosis in the PFC. A. Rats were treated as described in the legend of Figure 4. Pre- and Post-administration of Δ9-THC attenuated the astrogliosis induced by METH (Pre: −34% and −37%, Post: −47% and −29%, for 1 and 3 mg/kg, respectively) compared to control groups. **p<0.01 vs PRE METH-VEH and **^#^** *p*<0.05, and **^###^** *p*<0.001 vs POST METH-VEH (Bonferroni's post-hoc test). Horizontal dot lines represent the values of percentage of GFAP-IR density (1.31±0.10) in SAL-VEH group. B. Representative images of GFAP immunostaining in the PFC 72 h after the last METH or SAL administration in SAL-VEH, METH-VEH, METH- Δ9-THC 1 and 3 mg. Scale bar  =  100 µm.

With regard to the PFC (Figure 6), a two-way ANOVA revealed a significant main effect of treatment [F_(2,32)_ = 25.49, *p*<0.0001], as both doses of Δ9-THC significantly (*p*<0.001 vs METH-VEH, Bonferroni test) decreased GFAP-IR, while neither time [F_(1,32)_ = 0.22, *p* = 0.638] nor treatment x time interaction [F_(2,32)_ = 1.42, *p* = 0.254] were observed.

Data analyzed separately for time of treatment (pre- and post-METH administration) by one-way ANOVA revealed a lower GFAP-IR in both pre- and post-Δ9-THC treated rats than in controls. Specifically, both pre- and post-Δ9-THC-treated animals displayed a significant decrease of METH-induced GFAP-IR (PRE: -34% and -37%, for 1 and 3 mg/kg, respectively, *p*<0.01; POST: −47% and −29%; p<0.001 and p<0.05, respectively) as compared to their respective controls (Figure 6).

### Effects of SR on nNOS expression and GFAP-IR in the CPu and PFC

To determine whether the CB1 receptor was involved in the effect of Δ9-THC on nNOS overexpression and GFAP-IR, we tested the effect of the CB1 receptor antagonist SR on the lower dose of Δ9-THC tested given post-METH administration. When administered alone to SAL-treated rats, SR did not alter nNOS expression in the CPu (SAL-VEH = 31.36±1.04; SAL-SR = 27.00±1.40), nor GFAP-IR in the CPu (SAL-VEH = 0.75±0.08; SAL-SR = 0.66±0.04), nor GFAP-IR in the PFC (SAL-VEH = 1.30±0.10; SAL-SR = 1.03±0.17).

**Figure 7:**
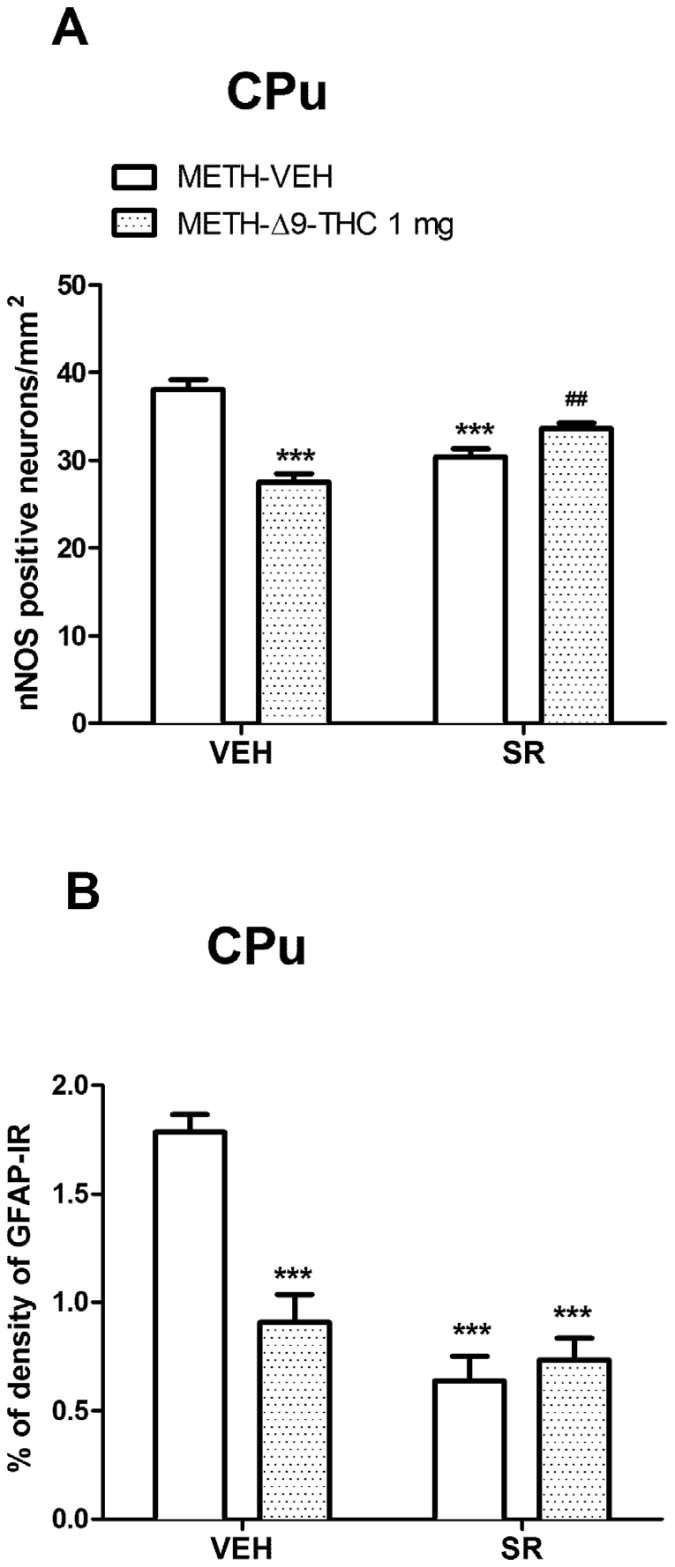
Effects of SR on nNOS and GFAP-IR in the CPu. A. Rats received injections of 1/kg Δ9-THC or VEH at 0.5, 12, 24, 36 and 48 h after the last METH administration (Post-treatment, POST) and were sacrificed 3 days after the last METH injection. SR (1 mg/kg, i.p.) or VEH were administered 15 min before each Δ9-THC or VEH injection. Two-way ANOVA in the CPu (A) showed a significant Δ9-THC x SR interaction (F_(1,40)_ = 32.45, *p*<0.0001); the administration of SR blunted the effect of Δ9-THC on METH-induced nNOS over-expression. SR alone decreased nNOS labeled neurons compared to that of control. ***p<0.001 vs METH-VEH (VEH pretreated) and **^##^**p<0.01 vs METH-VEH-Δ9-THC (VEH pretreated). B. Two-way ANOVA for GFAP-IR revealed a significant interaction between Δ9-THC and SR in the CPu (F_(1,35)_ = 19.86, *p*<0001). Δ9-THC and SR, alone or in combination, attenuated the METH-induced increase of GFAP-IR in the CPu. ***p<0.001 vs METH-VEH (VEH pretreated).

As shown in Figure 7A, regarding nNOS expression in rat pre-treated with SR, two-way ANOVA showed a significant interaction between the two factors (Δ9-THC and SR; F_(1,40)_ = 32.45, *p*<0.0001). A *post-hoc* analysis with Bonferroni test revealed that in the CPu of METH-Δ9-THC post-treated rats, nNOS staining was significantly weaker than in the METH-VEH treated group (*p*<0.0001). The number of nNOS positive neurons was significantly (*p*<0.01) higher in METH-SR-Δ9-THC than in METH-VEH-Δ9-THC group, indicating that SR slightly attenuated the Δ9-THC effect. Unexpectedly, SR by itself produced a significant (p<0.001) decrease of nNOS labeled neurons as compared to that of control.

As concerns GFAP-IR, two-way ANOVA revealed significant effects of Δ9-THC [F_(1,35)_ = 12.70, *p* = 0.001] and SR [F_(1,35)_ = 36.49, *p*<0.0001] treatment in the CPu, along with a Δ9-THC x SR interaction [F_(1,35)_ = 19.86, *p*<0.0001]. Bonferroni *post-hoc* test showed that both drug treatments, alone or in combination, significantly (*p*<0.001) reduced METH-induced GFAP-IR (Figure 7B).

**Figure 8:**
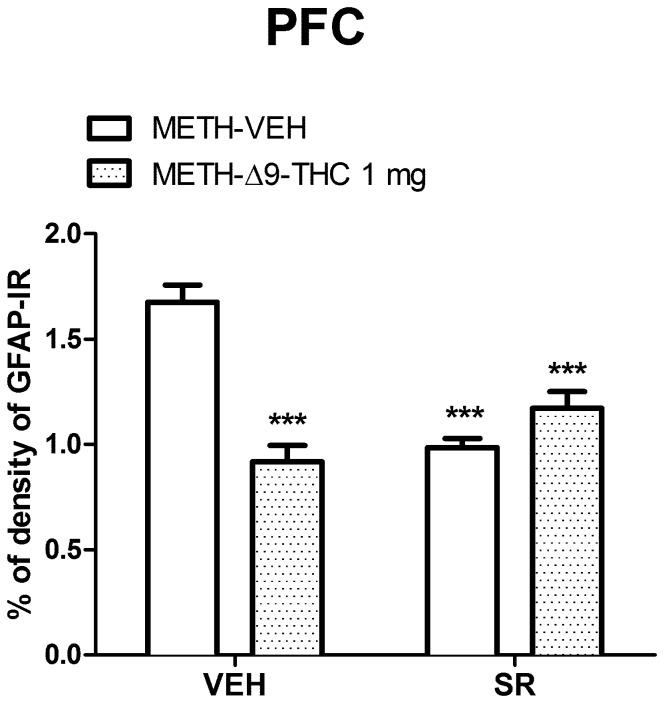
Effects of SR on GFAP-IR in the PFC. Two-way ANOVA for GFAP-IR revealed a significant interaction between Δ9-THC and SR in the CPu (F_(1,33)_ = 45.91, *p*<0001). METH-Δ9-THC significantly reduced METH-induced GFAP-IR. Moreover, GFAP-IR was lower in METH-SR-VEH and METH-SR-THC groups as compared to METH-VEH treated rats. ***p<0.001 vs METH-VEH (VEH pretreated).

In the PFC (Figure 8), a significant effect on GFAP-IR was detected for Δ9-THC (F_(1,35)_ = 12.02, *p* = 0.0014] and SR treatment [F_(1,35)_ = 7.09, *p* = 0.011], as well as for Δ9-THC x SR interaction (F_(1,35)_ = 32.88, *p*<0.0001]. GFAP-IR was lower (*p*<0.001, Bonferroni *post-hoc*) in METH-Δ9-THC (1 mg/kg) post–treated than in METH-VEH rats. Moreover, SR alone or in combination with Δ9-THC significantly reduced (*p*<0.001, Bonferroni *post-hoc*) GFAP-IR compared to controls.

These results suggest that Δ9-THC (1 mg/kg) decreases METH-induced nNOS over-expression and GFAP-IR via a CB1 receptor-dependent and independent mechanism, respectively.

## Discussion

In the present study, we showed that Δ9-THC, the principal constituent of cannabis, attenuates the neurotoxic effect of METH by reducing two markers of neuronal damage, overexpression of nNOS and astrogliosis. Specifically, METH-induced astrogliosis and nNOS overexpression were reduced by pre- and post-treatment with Δ9-THC in the CPu and PFC, respectively.

NO plays a key role in METH-induced neurotoxicity [7], [8]. NO is a free radical gas, and highly reactive molecule, that functions as a neurotransmitter or neuromodulator, when synthesized by the enzyme, nNOS [38], and is an important mediator in a variety of central nervous system disorders, including METH-induced neurotoxicity. The increase in extracellular glutamate caused by neurotoxic doses of METH activates NMDA receptors, resulting in increased intracellular Ca^2+^ that leads to activation of nNOS via, a Ca^2+^-calmodulin-dependent mechanism, and production of NO. METH-induced neurotoxicity is prevented by co-administration of NOS inhibitors [39]; the neuroprotective effect of these inhibitors might also involve the reduction of METH-induced hyperthermia [40]. Several studies have described interactions between cannabinoids and NOS, indicating that the neuronal CB1 receptor is involved in the regulation of NO synthesis. Indeed, cannabinoids prevent NO-mediated neurotoxicity of retinal neurons [41] and protect neurons from NMDA toxicity *in vitro* and *in vivo* through a mechanism that involves the activation of nNOS and protein kinase A [42]. Notably, nNOS activity in the cerebral cortex is higher in CB1 receptor knockout than in wild-type mice [42].

METH-induced increases in extracellular glutamate also leads to astrocyte activation; this activation leads to the release of pro-inflammatory cytokines that stimulate glutamate release and inhibit glutamate uptake [43] which increases NOS synthase activity and ROS production, eventually causing neuronal damage [8]. Repeated *in vivo* METH treatment induces a significant increase of GFAP levels in the striatum, cortex, and hippocampus [6]. Anti-inflammatory drugs (i.e. ketoprofen, indomethacin, tetracycline, and minocycline) protect against METH-induced gliosis and neurotoxicity [44], [45].

In the present study, we found a significant increase in the number of positive nNOS neurons and GFAP immunostaining in the CPu and PFC of METH-treated rats. These data confirm the involvement of nNOS and astrocytes activation in METH-induced neurotoxicity [6], [29]. The findings of hyperthermia and the pattern of nNOS and GFAP immunostaining observed in the present study are consistent with those reported previously [3], [29], which support our proposed model of METH-induced neurotoxicity. The validity of our model of METH neurotoxicity is further strengthened by the finding that a METH dose lower than that we used here (4 mg/kg) administered accordingly to the same “binge” schedule (4 administrations, 2 hours apart) is able to induce toxicity on 5-HT and DA innervations. Indeed, 3 and 7 days following the last METH administration we reported a loss of DAergic and 5-HTergic terminals measured by means of immunohistochemical detection of their transporters (5-HTT and DAT) [46]. Moreover, this model of METH administration (4×10 mg/kg, 2 h apart) is currently the most frequently used rat model of METH neurotocivity, and is associated with striatal dopamine and serotonin depletion, hyperthermia and high mortality [7]. However, other studies have reported no differences nNOS expression [47], a discrepancy likely due to differences in animal species and strains [48] as well as procedural differences, such as drug doses, METH administration schedule, and time intervals between drug treatment and immunohistochemical studies. Interestingly, pre- and post-treatment with Δ9-THC significantly decreased the overexpression of striatal nNOS and METH-induced gliosis in the rat PFC and CPu, suggesting a neuroprotective effect of cannabinoid agonists likely mediated, at least in part, by their anti-inflammatory properties. Cannabinoid agonists have been shown to inhibit NO in microglia, neurons, and macrophages [49]. METH-induced neurotoxicity and THC exposure are associated with hyperthermia [3], [8] and hypothermic effects, respectively. We therefore tested the effects of Δ9-THC on METH-induced neurotoxicity, but contrary to previous findings [36] showing a decreasing effect of Δ9-THC on NMDA-induced hyperthermia, in our study pre-treatment with Δ9-THC failed to prevent METH-induced hyperthermia. This suggests that the observed Δ9-THC neuroprotection is temperature-independent.

In this study, we choose to use multiple rather than chronic Δ9-THC treatment to avoid negative emotional states (e.g., anxiety, depression, lack of motivation) [50], [51], and the reduction in the white and gray matter in the cerebellum often described in chronic cannabis users [52], [53]. Animal studies have reported long-lasting cognitive and memory deficits following chronic Δ9-THC exposure [54], [55], as well as neuronal death and reduced synaptic density of pyramidal neurons in the hippocampus [55], [56]. Δ9-THC doses used in this study are within the range of doses that have been shown to induce neuroprotective effects [11], [35], [36].

The lack of dose-response of the attenuating effect of Δ9-THC on METH-induced nNOS overexpression and astrogliosis suggests that the maximal level of neuroprotection might have been obtained at 1 mg/kg of Δ9-THC (ceiling effect). Notably, an intraperitoneally administration of 0.002 mg/kg has been found to induce long-term neuroprotection after repeated administration of MDMA [11], [57]. This finding has been attributed to the pre- and post-conditioning phenomena, in which a minor noxious stimulus (Δ9-THC) protects a subsequent or preceding insult (neurotoxicity). Thus, we cannot exclude that the protective effect of Δ9-THC observed in our study could also be obtained with lower doses [57]. Therefore, future studies will evaluate whether lower doses can induce Δ9-THC-mediated neuroprotection. Moreover, our data showing that post-treatment 3 mg/kg THC had less effect than 1 mg/kg THC on GFAP-IR were completely unexpected. At the moment we don't have any plausible hypothesis to explain these findings.

Microglial cells and CB2 receptors are also likely to play a role in the neuroprotective effects of Δ9-THC on METH-induced neurotoxicity observed in this study. Cannabinoid CB2 receptors are present in both microglia and astrocytes [58], and their activation mediates immunosuppressive effects, limits inflammation, and is associated with tissue injury under several pathological conditions, including those associated with neurodegeneration [59]. Repeated administration of the CB2 receptor agonist JWH-105 reduces the inflammatory response to MDMA and provides partial protection against 5-hydroxytriptamine neurotoxicity [60]. Stimulation of CB2 signaling elicits a series of molecular and cellular events that attenuates delayed neurodegeneration [34]. Future studies should be performed in order to evaluate the potential role of CB2 receptors in both neurons and microglia in THC-induced neuroprotection.

Finally, we pretreated rats subjected to METH and Δ9-THC post-treatment with the CB1 receptor antagonist SR to determine whether Δ9-THC inhibition of METH-induced nNOS overexpression and gliosis occurred through a CB1-mediated mechanism. In the CPu, SR attenuated the neuroprotective effect of Δ9-THC on METH-induced nNOS overexpression. This effect is most likely due to action on either CB1 receptors located presynaptically in glutamatergic terminals or on astrocytes, which could result in increased glutamate excitoxicity. With regard to METH-induced astrogliosis, SR did not revert the decreasing effect of Δ9-THC on METH-induced GFAP-immunostaining both in the striatum and PFC. These findings suggest that Δ9-THC-mediated inhibition of METH-induced astrogliosis is likely to occur through a CB2-receptor dependent mechanism, as recently reported for the suppression of MDMA-induced astrocytes activation [36].

Unexpectedly, we found that SR suppressed METH-induced astrogliosis in both brain areas, an effect that to our knowledge has not been described previously. SR has been reported to exert neuroprotective effects in animal models of cerebral ischemia, trauma, and neuronal damage induced by NMDA [61], [62]. In animal models of cerebral artery occlusion, SR was found to exert a neuroprotective effect which was associated with (i) an increase in the striatal content of anandamide (AEA), (ii) an enhanced activity of N-acylphosphatidylethanolamine-hydrolyzing phospholipase D, and (iii) reduced expression and activity of fatty acid amide hydrolase (FAAH) [63], [64], [65]. A possible role for the transient receptor potential vanilloid 1 (TRPV1) on the neuroprotective effect of SR has been suggested by Pegorini *et al.* 2006 [66] who demonstrated that the neuroprotective effect shown by SR in an animal model of transient forebrain ischemia was prevented by the TRPV1 antagonist capsazepine. These findings suggest that SR may protect against excitotoxicity by blocking CB1 receptors and preventing their activation by the endogenously generated AEA, which accumulates during brain injury [67]. Since AEA activates, although with different affinity, both CB1 and TRPV1 receptors [68] and up-regulates genes involved in pro-inflammatory related responses [69], the increased concentration of AEA activates and desensitizes the TPRV1 [66], inducing a neuroprotective effect. Moreover, N-acyl-phosphatidylethanolamine (NAPE) and N-acylethanolamine (NAE), including AEA, are produced in neurons in response to the high intracellular Ca^2+^ concentrations that occur in injured neurons [70].

As glutamate excitotoxicity is one of the mechanisms through which METH induces neurotoxicity, in our model, SR protects against METH-induced neurotoxicity by signaling the increased accumulation of AEA to TRPV1 receptors, leading to desensitization and inducing a neuroprotective effect. Alternatively, the effect of SR on glutamate release may be mediated by a CB1-independent mechanism, as reported *in vitro* in hippocampal synaptosomes of rats and mice [71].

In conclusion, although comorbid cannabis and METH use might worsen mental health problems in drug users [72], this study provides the first evidence that Δ9-THC reduces METH-induced brain damage via inhibition of striatal nNOS expression by both CB1-dependent and -independent mechanisms and of striatal and cortical astrocyte activation by CB1-independent mechanisms only.
